# Human Papillomavirus-Related Cutaneous Squamous Cell Carcinoma

**DOI:** 10.3390/cancers17050897

**Published:** 2025-03-05

**Authors:** Alejandra Sandoval-Clavijo, Ignasí Martí-Martí, Carla Ferrándiz-Pulido, Júlia Verdaguer-Faja, Ane Jaka, Agustí Toll

**Affiliations:** 1 Department of Dermatology, Hospital Clínic de Barcelona, 08036 Barcelona, Spain; 2 Faculty of Medicine, Universitat de Barcelona, 08036 Barcelona, Spain; 3 Department of Dermatology, Hospital Universitari Vall d’Hebron, 08035 Barcelona, Spain; 4 Faculty of Medicine, Universitat Autònoma de Barcelona, 08035 Barcelona, Spain; 5 Department of Dermatology, Hospital Universitari Germans Trias i Pujol, 08916 Badalona, Spain

**Keywords:** squamous cell carcinoma, cutaneous squamous cell carcinoma, human papillomavirus, epidermodysplasia verruciformis, keratoacanthoma, Bowen’s disease

## Abstract

The human papillomavirus (HPV) has been associated with the carcinogenesis of cutaneous squamous cell carcinoma (cSCC), especially in immunosuppressed patients. This article reviews the microbiology of HPV and its role in tissue tropism, invasion, and oncogenesis. It also describes possible HPV oncogenic ability due to the inactivation of the host p53 and retinoblastoma protein (pRb) by HPV oncoproteins E6 and E7, producing a suppression of cell cycle checkpoints and uncontrolled cell proliferation that may eventually result in invasive carcinoma. We will focus on β-HPV types and their role in epidermodysplasia verruciformis (EV), as well as α types and their ability to cause cutaneous and mucosal pathology. We also intend to examine the clinical characteristics of cSCC related to HPV and host immunosuppression conditions such as solid organ transplant in order to provide management guidelines for patients with cSCC associated with HPV based on available data. Other topics addressed in this article include particular locations of cSCC, such as nails; the prognosis; the recurrence; therapeutic modalities; and the role of HPV vaccines.

## Simple Summary

Cutaneous squamous cell carcinoma (cSCC) is the second most prevalent subtype of skin cancer, particularly among elderly patients. There are multiple factors associated with the carcinogenic development of this type of tumor including the human papillomavirus (HPV), particularly in immunocompromised patients. This article reviews the role of human papillomavirus (HPV) in the oncogenesis of cSCCs as well as their clinical characteristics, prognosis, and therapeutic approaches.

## 1. Introduction

Cutaneous squamous cell carcinoma (cSCC) is the second most frequent nonmelanoma skin cancer, accounting for 20% of these tumors in the U. S and showing an increasing trend in incidence rates. The prevalence of cSCC varies depending on geographic regions, demographics, and environmental factors with an age-standardized incidence ranging from 9 to 96 per 1,000,000 male inhabitants and 5 to 68 per 100,000 female inhabitants in Europe [1]. The human papillomavirus (HPV) may trigger the development of muco-cutaneous squamous cell carcinomas (cSCCs), especially in immunosuppressed patients and in tumors of the oro-genital area. We herein review the microbiology of HPV and the genera that show tissue tropism and oncogenic ability. We will focus on β-HPV types and their role in epidermodysplasia verruciformis (EV), as well as α types and their ability to cause cutaneous and mucosal pathology. We also examine the clinical characteristics of cSCC related to HPV and host immunosuppression conditions such as solid organ transplant in order to provide management guidelines for patients with cSCC associated with HPV based on available data. Other topics addressed in this article include particular locations of cSCC, such as nails; the prognosis; the recurrence; therapeutic modalities; and the role of HPV vaccines.

## 2. Microbiology and Carcinogenesis

**Figure 1:**
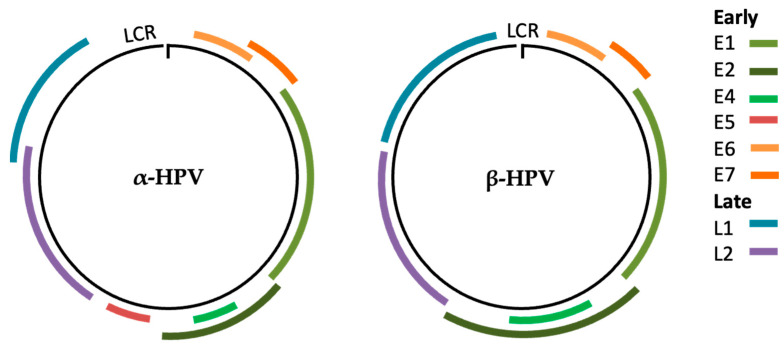
Comparative genome arrangement of α and β genera. Despite having a similar genetic structure, the size and position of the major ORFs can vary; also, β HPV lacks E5 ORF.

Human papillomaviruses (HPVs) are non-enveloped viruses that contain an approximately 8 kb circular DNA genome [2]. This genome is composed of an upstream regulatory region (URR), an intergenic noncoding region (NCR) with simple (AT)n and poly-T repeats, and eight main expressed protein-coding open reading frames (ORFs) [3]. The ORFs are so called according to their expression time during the viral life cycle, where the letters “E” and “L” stand for early and late, respectively [3]. ORFs expressed across different viral cell cycle phases and carcinogenesis processes are E1, E2, E4, E5, E6, E7, L1, and L2. All alpha HPVs share this arrangement (Figure 1). However, all known HPVs have in common four ORFs (E1, E2, L1, and L2), required to trigger viral replication and shedding [4,5]. Moreover, certain HPV genera lack an ORF, such as E5, which is not present in beta, gamma, and mu genera, thus suggesting that the protein can provide some additional but not essential value, such as promoting infection and transformation [5,6].

More than 200 types of HPV have been described [7]. All of them replicate within stratified squamous epithelial cells, although they vary in their capacity to infect cutaneous or mucosal keratinocytes. The classification of papillomaviruses is according to the guidelines from the International Committee on the Taxonomy of Viruses [8], and they are grouped into the same genus (>60% identity), species (>70% identity), and type (>90% identity) based on an empirical distribution of pairwise L1 nucleotide sequence identity [3,8].

Five major genera of HPV that infect humans have been described: α (alpha), β (beta), γ (gamma), μ (mu), and υ (nu). These genera differ in their tropism, which is determined by viral entry capacity by the interaction of the L1 capsid protein with the host cell surface [9,10]. HPVs that infect mucosal keratinocytes are subdivided into low-risk types, which cause benign lesions such as condylomas or warts, and high-risk types, which are those related to malignant neoplasms such as cervical cancer [3,11,12,13]. Currently, 12 HPV types of α genera have been linked to carcinogenesis: types 16, 18, 31, 33, 35, 39, 45, 51, 52, 56, 58, and 59 [3].

The invasion of the HPV is a complex process that requires, firstly, the entrance through the tissue surface via small epithelium damage to reach the basal epithelial cells. Then, capsid proteins, L1 (major coat protein) and L2 (minor coat protein), facilitate the entry into the basal layer keratinocytes through the interaction with heparan sulfate proteoglycan (HSPG) [7,14]. L2 also allows the access of the viral genome to the host cell nucleus, where the replication process begins via proteins E1 and E2, as well as the host cell’s replication machinery [15,16].

After HPV succeeds in basal cell invasion, the HPV DNA integration into host DNA is caused by the alteration of the E1/E2 open reading frames of the HPV genome and subsequent deletion of the E2-controlled regulation of E6 and E7. The early ORF proteins E6 and E7 develop a major role as HPV oncoproteins, promoting the cell cycle re-entry and proliferation of HPV-infected cells; they reduce the expression of p53 and retinoblastoma proteins (pRb), inhibit cell differentiation, enhance DNA replication, and elude host defenses [2,3]. This ultimately leads to a suppression of cell cycle checkpoints and uncontrolled cell proliferation that may eventually lead to invasive carcinoma [13,17,18]. Moreover, HPV may also stimulate host immune evasion mechanisms [19].

## 3. Mucosal and Periungual SCCs Are Associated with α-HPVs

HPV infection is responsible for a large subset of carcinomas in the lower anogenital tract and for approximately 25% of the head and neck carcinomas, mainly due to α-HPV [20,21,22,23,24].

### 3.1. Oral Cavity

Oral cavity squamous cell carcinoma (OCSCC) is one of the most frequent cancer subsites in the head and neck area [25], and originates from the epithelial cells of the mobile tongue, floor of the mouth, hard palate, buccal mucosa, or gingivae [26]. Most relevant risk factors for OCSCC are tobacco consumption and alcohol, with an RR of 5.8 for heavy smokers and an RR of 7.4 for drinkers. Nonetheless, the effect of alcohol and tobacco joint consumption proved to be synergistic, multiplying the risk and showing an RR of 37.7 for a simultaneous drinker and smoker [27]. Other well-known risk factors for OCSCC are poor oral hygiene, some hereditary syndrome such as Fanconi anemia and dyskeratosis congenita, or immunosuppression [26]. Although it is well established that HPV is an important risk factor for oropharyngeal squamous cell carcinomas (OPSCCs), its role in OCSCC remains controversial [26,28].

There is a significant heterogeneity in the literature regarding the prevalence of HPV-positive OCSCC, ranging from 0% to 37% of cases (I2 > 75%, *p* < 0.01). However, a recent meta-analysis from Katirachi et al. revealed a worldwide prevalence of HPV-positive OCSCC of 6% (95% CI; 3–10%), and a bigger proportion of HPV-negative OCSCC than HPV-positive OCSCC in all the studies. This suggests that HPV infection is not a necessary nor a strong risk factor for OCSCC development, and might contribute to a low proportion of OCSCC worldwide [26,28].

The most prevalent HPV genotype is HPV-16, followed by HPV-18, and less frequently HPV types 31, 33, 45, 52, and 59 [26,28].

It is more common in men, and located on the ventral tongue and floor of the mouth [29]. HPV-positive OCSCC occurs in older individuals, and is more frequently associated with tobacco or alcohol consumption [26,29]. In addition, a higher alcohol consumption and the number of sexual partners were demonstrated to be associated with HPV-positive OCSCC in one study [30]. This is in contrast to HPV-positive OPSCC, which shows as being more apparent among younger patients with less use of alcohol or tobacco [30]. 

Histologically, the main phenotypes of HPV-positive tumors in the oral cavity are the nonkeratinizing basaloid and the warty morphologies, while only 10% of cases exhibit the conventional keratinizing features. This warty subtype showed a trend toward better outcomes. Of note, a distinctive HPV-related intraepithelial proliferation can be found around OCSCC [29,31].

While HPV infection and p16 overexpression are a widely recognized cause and favorable prognostic factor for OPSCC [32], the latest evidence supports that neither p16 nor HPV status have an impact on OCSCC survival [26,29,33]. Although still unclear, this difference may be explained as the oncogenicity of HPV infection is of less magnitude in the pathogenesis of OCSCC, while other risk factors such as alcohol and tobacco play a major role [28]. In addition, although HPV16 is the predominant type associated with OPSCC, in OCSCC, other HPV types may also be present, and studies that focus exclusively on HPV16 may therefore underestimate them [33].

### 3.2. Vulvar and Penile SCC

Overall, 39.1% of vulvar carcinomas and 46.9% penile carcinomas are HPV-attributable [21,34]. The most predominant HPV genotype in vulvar carcinomas is HPV-16, followed by HPV-33 [34], while in penile carcinomas, the most common subtype is HPV-16, followed by HPV-18 and in <10%, HPV-6/11 [35]. However, this relatively high proportion of HPV-6/11 may be explained as a co-infection with other high-risk subtypes and not necessarily causative for carcinoma [35].

Persistent HPV infection, especially high-risk subtypes, may induce intraepithelial neoplasia, which with time can progress to invasive carcinoma. This process is driven by the oncoprotein E6 and E7 expression, which promote p53 and Rb pathway inactivation [21]. Certain similar traits of HPV-related disease justify a common classification for all lower anogenital tract squamous intraepithelial lesions (SILs): low-grade (LSIL) and high-grade (HSIL) [21,36]. LSIL represents the morphologic manifestation of transient HPV infection with a high rate of regression, while HSIL represents the persistence of high-risk HPV infection and viral integration with a significant rate of progression to invasive carcinoma. Despite certain common traits, SILs encounter unique diagnostic challenges by anatomic site. In general terms, LSIL histological features consist in irregularly dispersed, enlarged hyperchromatic nuclei with cytoplasmic halos in upper layers, the lower third without significant squamous maturation, and mitotic figures usually limited to lower layers. On the contrary, HSIL shows a crowded proliferation of atypical basaloid cells with hyperchromatic irregular nuclei, a minimal squamous maturation, and dyskeratotic cells, and mitotic figures may be found in all the layers of the epithelium [21].

Clinically, they present as irregular, sharply demarcated, slightly palpable lesions, with white to red or brown pigmentation, and commonly associated pruritus [37].

HPV-associated vulvar and penile carcinomas typically arise in younger individuals, adjacent to an intraepithelial neoplasia, and associated with risk factors related to sexual practices. Basaloid and warty squamous cell carcinomas are the most frequent histological subtypes associated with HPV infection [35,38].

The prognostic value of HPV status in vulvar and penile carcinomas remains unclear. However, p16 positivity has been associated with a favorable prognosis [38,39], and there is some evidence suggesting increased radiosensitivity of HPV-associated vulvar and penile carcinomas [38,40].

### 3.3. Anal SCC

**Figure 2:**
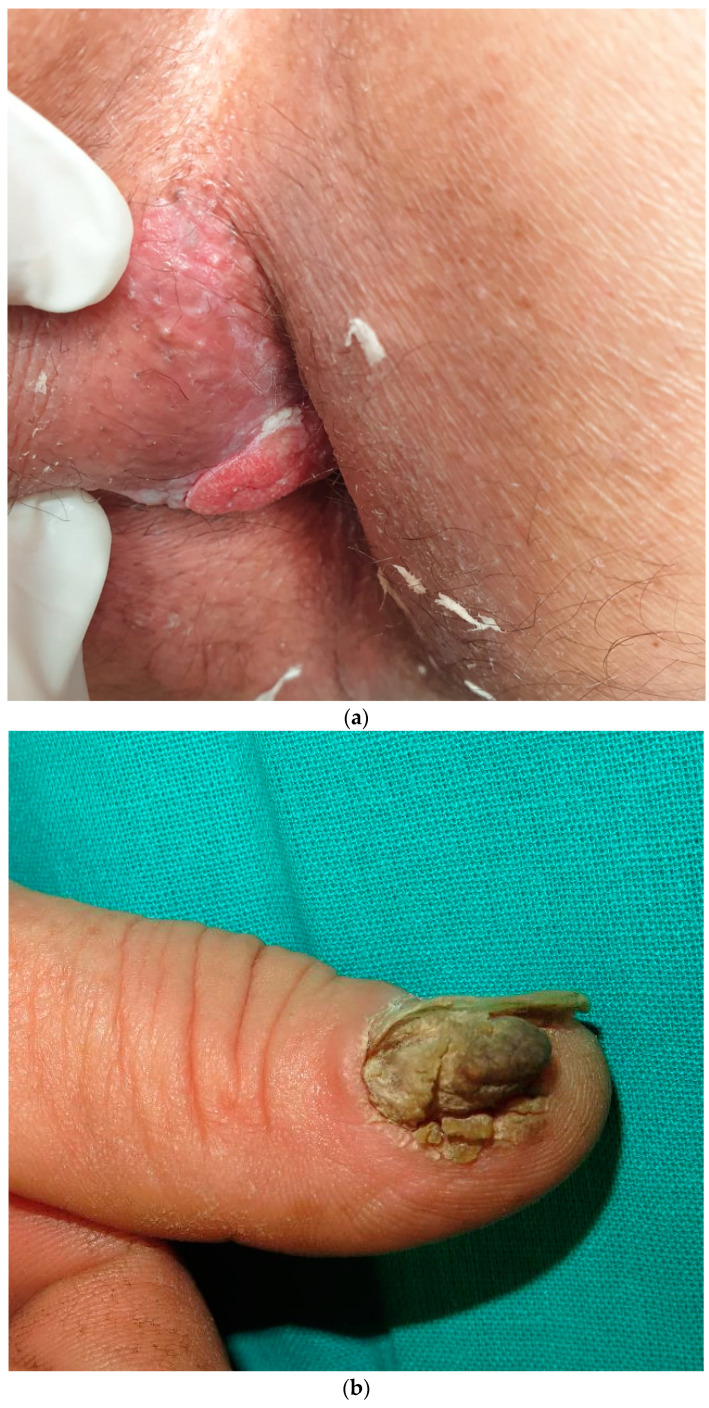
Clinical appearance of HPV-associated SCC. (**a**) Plaque HPV 16+ in the perianal region, corresponding to in situ SCC. (**b**) An infiltrative, verrucous plaque in the periungual region with nail plate destruction.

Around 90% of anal squamous cell carcinoma (ASCC) can be attributed to HPV, more commonly HPV16, and secondly HPV18 (Figure 2a). Histologically, there is a clear squamous differentiation [13,36,41].

There are specific groups with high risk of developing ASCC, namely HIV-positive patients, men who have sex with men, women with a previous history of genital tract neoplasia, and solid organ transplant recipients [36]. Contrary to cervix carcinoma, it is still not clear whether HIV-induced immunosuppression differentially affects the potential of HPV to induce neoplastic changes in the anus [42]. Moreover, the fraction of ASCC attributable to HPV16 seems to be smaller in the HIV-positive population, compared to the HIV-negative population with ASCC [42]. On the other hand, highly active antiretroviral therapy (HAART) has not been shown to be associated with lower ASCC rates in HIV-positive patients. In fact, the incidence of ASCC during the post-HAART period is higher, regardless of CD4 count, probably due to longer survival of patients [36].

The HPV/p16 positivity seems to have a favorable prognostic value in ASCC, being associated with better systemic treatment response, and improvement in both the overall survival and cancer-related survival rate [43,44].

Patients with condylomas, despite being caused by low-oncogenic-risk HPV, have a higher risk of developing subsequent HPV-related cancer, probably due to coexisting risky sexual behaviors that may lead to reinfection by other HPV subtypes [13,45]. In addition, HPV-anogenital-cancer survivors seem to have an increased risk of HPV-associated second primary malignancies, probably since HPV infections are often multifocal [46,47].

### 3.4. Periungual SCC and HPV

Squamous cell carcinoma is the most common cancer of the nail. In this particular location, the presence of α HR-HPV is detected in approximately half of tumors [48,49,50]. Other types of HPV seem to play a minor role. The most frequently detected genotype is α-HPV type 16 [48,49].

A review found that one out of four patients with nail SCC have other HR-HPV-associated diseases. Moreover, two case series studies found a correlation through DNA sequencing of the virus between genital dysplastic lesions and the presence of the virus in the nail or finger [51,52]. Therefore, a genito-digital mode of transmission has been suggested [48,49].

Clinical presentation is usually a periungual verrucous papule or a subungual hyperkeratosis leading to onycholysis and nail plate destruction (Figure 2b). Other changes, such as longitudinal erythronychia, may be observed [53]. In any case, a clear diagnostic delay has been reported with half of the cases being misdiagnosed and treated improperly [48,49]. It is probably due to the fact that it can resemble many other benign inflammatory processes such as periungual warts or onychomycosis, which are much more common. Therefore, its diagnosis requires a high level of suspicion. Tissue destruction and the involvement of a single finger would favor the diagnosis of carcinoma [48,49].

This disease predominantly affects men at a younger age compared with other cSCC [48]. A recent study by our group found that HR-HPV-associated SCC of the nail appears in younger patients, and is less infiltrative and probably more recurrent than those not associated with HR-HPV [49]. Although there is no high-level scientific evidence, Mohs surgery seems to be more effective in treating these lesions than conventional surgery. Given the high recurrence rate compared to other locations, even when using this technique, a closer follow-up may be recommended [48,49].

## 4. Epidermodysplasia Verruciformis and cSCC Developing in Immunosuppressed Patients

The first evidence for the existence of human papillomavirus types with cutaneous tropism (beta-human papillomaviruses (b-HPVs)) cooperating with ultraviolet (UV) radiation in the development of keratinocyte carcinoma (KC) was reported in 1922 in patients with epidermodysplasia verruciformis (EV) [54].

EV is a rare genetic skin disorder characterized by a high susceptibility to infection by b-HPVs, which are considered to be harmless for the general population. The prevalence of EV is less than 1 in 1,000,000 people [54]. This genetic disorder is inherited in an autosomal recessive pattern and persists throughout life, often leading to the development of KC in sun-exposed areas [55].

EV can be either genetic or acquired. Genetic EV is caused by mutations in the transmembrane channel genes *EVER1/TMC6* and *EVER2/TMC8*, present in over 75% of cases. Mutations in other genes such as *RHOH*, *MST1*, *CORO1A*, or *ECM1* have also been described but a considerable number of clinically diagnosed EV patients exhibit no identifiable genetic alterations [56,57,58,59,60,61,62]. All mutations are loss-of-function mutations and result in a lack of protein production [54]. Acquired EV, associated with HIV infection or iatrogenic immunosuppression such as solid organ transplant recipients (SOTRs), lacks these genetic mutations but shares a similar clinical presentation [63].

**Figure 3:**
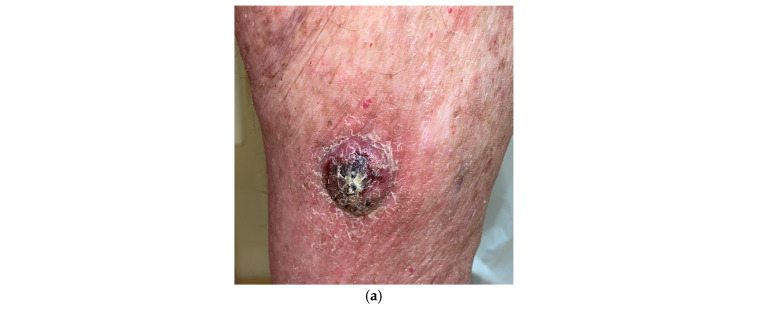

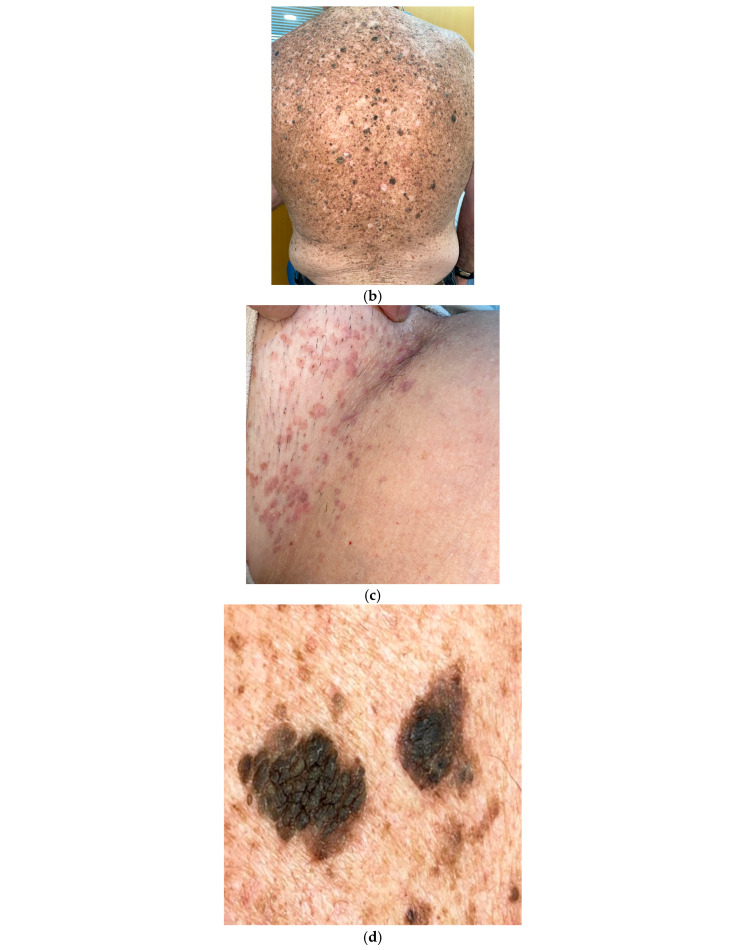

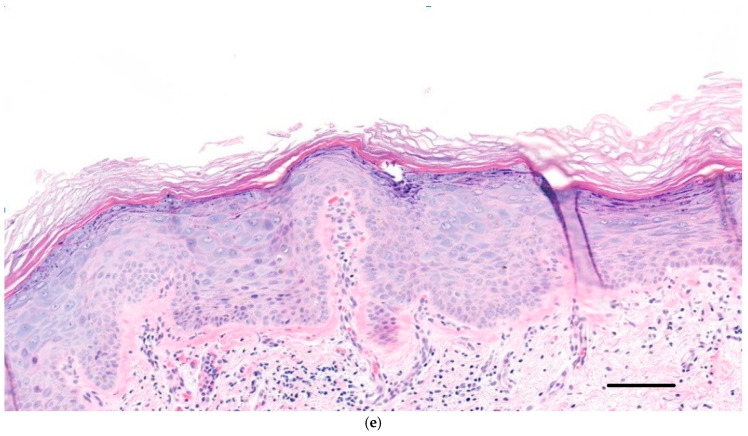
Clinical appearance of different tumors in patients with EV phenotypes. (**a**) Rounded hyperkeratotic cSCC, with well-defined borders, in the leg of a renal transplant patient with an EV phenotype. (**b**) Multiple verruco-keratous lesions in a renal transplant patient undergoing treatment with AZA with an EV phenotype. (**c**) Flat warts in a lung transplant patient with acquired EV. (**d**) Seborrheic keratosis with a hyperkeratotic verrucous surface. (**e**) Histopathology of flat warts in a lung transplant patient with acquired EV showing epidermal hyperkeratosis, orthokeratosis, mild papillomatosis, acanthosis, and viral inclusion bodies (scale bar = 100 µm H&E, 10×).

Patients affected by EV typically develop during infancy flat hypo- and hyperpigmented macules in the trunk, neck, and limbs, which may evolve to verruco-keratous lesions, papillomas, seborrheic keratoses, and reddish pityriasis versicolor. These lesions can progress to KC, among all cutaneous squamous cell carcinoma (cSCC), particularly in the UV-exposed areas [64]. This transformation from healthy skin to malignancy could take approximately 20 years. In the presence of multiple verruco-keratotic lesions, it can sometimes be difficult to identify the lesion that has already undergone malignant transformation and has become infiltrating cSCC (Figure 3a). Even microscopically, benign lesions can show architectural changes with epidermal hyperplasia, cellular changes suggestive of viral infection, and malignant transformation, which can make it difficult to distinguish a viral wart from a seborrheic keratosis or cSCC (Figure 3b–e). The difficulty in distinguishing between these lesions can lead to a delayed diagnosis and treatment of cSCC.

While HPV-5 and 8 are the ones most commonly associated with EV-related cSCC, other β-HPV types may also contribute. The more serotypes identified in a patient, the greater the risk of developing KC. Moreover, the diversity and load of β-HPV types in eyebrow hair are associated with cSCC risk in SOTRs, providing more evidence that β-HPV is associated with cSCC carcinogenesis and may present a target for future preventive strategies [65].

Mechanistic insights into β-HPV’s role in skin carcinogenesis are still not fully understood, but evidence suggests a synergistic interaction with UV radiation and immune suppression. The “hit-and-run” hypothesis tries to explain the role of β-HPV by the virus being necessary at an early stage of carcinogenesis, promoting the deleterious effects of UV radiation, but being dispensable for the maintenance of the malignant phenotype of KC, because they are not transcriptionally active in KC [65,66]. UV-induced mutations induce cell cycle arrest and apoptosis of keratinocytes in immunocompetent patients. In contrast, keratinocytes remain alive despite the accumulation of UV-induced DNA mutations in immunosuppressed patients infected with β-HPV. Thus, UV radiation is the main driver of skin cancer development, while beta HPVs act as facilitators of the accumulation of UV-induced DNA mutations [66,67]. Recent findings strongly suggest that the antiviral adaptive immune responses define the role of β-HPV in skin carcinogenesis. The β-HPV-specific CD8+ T cells are important for preventing skin cancer in immunocompetent individuals. Immunosuppression, however, appears to increase the risk of skin cancer not because HPVs are directly oncogenic, but because it weakens the immune system’s ability to control the activity of these viruses [66,67,68].

## 5. Cutaneous cSCC and HPV in Non-Immunosuppressed Patients, a Controversial Relationship

There is no definite consensus regarding the relationship between HPV and non-mucosal SCC [69,70,71]. Although the majority of studies report HPV infection in varying percentages of cSCC, with a higher prevalence in immunocompromised patients rather than immunocompetent ones, some research has not found any correlation between HPV and SCC [70,71]. The lack of consensus may be a consequence of significant variability in study results caused by different HPV types examined, sample methods used, and viral detection procedures.

However, the higher HPV prevalence in SCC compared to normal skin does not necessarily imply causality. The absence of HPV in some cSCC could indicate that the virus is involved in triggering the oncogenesis process rather than maintaining it [48,49]. According to a study that used type-specific real-time PCR for six frequent β-HPVs to determine viral load in various clinical lesions, precancerous lesions such as actinic keratoses had a higher viral load of HPV than primary SCC, metastatic tumors, or even perilesional skin [70,72].

HPVs may act as co-carcinogens with other factors, amplifying the risk of cSCC development. UV light has been shown to alter the function of keratinocytes and immune cells, triggering the release of proinflammatory cytokines and promoting cell proliferation and angiogenesis. Also, UV light may induce mutations in *TP53*, *HRAS*, and *Notch*, which regulate normal squamous cell differentiation [73]. HPV might alter the DNA repair process, inducing a major susceptibility to UV-induced damage [71].

**Figure 4:**
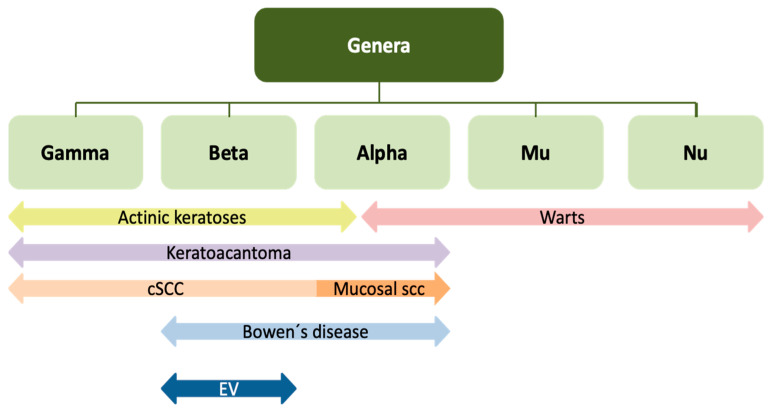
Graphical representation of types of HPV genera associated with mucosal and cutaneous lesions.

The specific link between β-HPV genera and SCC in immunocompetent patients is also up for debate [67]. However, the VIRUSCAN Study, which included viral DNA testing for 52 β-HPV and 46 γ-HPV types in immunocompetent patients, found an increased cSCC risk associated with β-HPV types, specifically with HPV-24 [74]. β and γ-HPV subtypes have mainly been found in actinic keratoses (AKs) and in invasive non-mucosal cSCC, differing from Bowen’s disease (BD), in which α and β are the predominantly found genera. On the other hand, keratoacanthoma (KA) usually presents α, β, and γ genera [19,75,76]. However, a few AKs might also be positive for α-HPV genera [19,76] (Figure 4). Heterogeneous HPV prevalence rates have also been found to be associated with different clinical–pathological SCC variants. Of these, keratoacanthoma exhibited the highest frequency (around 90%), while BD and SCC had lower frequencies (around 50%) [77,78].

## 6. Therapeutic Considerations in HPV-Related SCC

As mentioned above, HPV-associated SCCs often appear in anatomically complex areas such as genitalia and fingers and may manifest as multiple lesions that are difficult to treat. We comment on the possible therapeutic approaches in this subset of patients.

### 6.1. In Situ HPV-Related SCC

Destructive topical treatments such as imiquimod, topical retinoids, photodynamic therapy, cryotherapy, and 5-fluorouracil can be used for the treatment of congenital or acquired EV [79,80]. Nevertheless, when widespread lesions of EV are present, some authors recommend oral retinoids in a continuous systemic low-dose regimen [80].

Cryotherapy, topical agents (imiquimod, 5-fluorouracil), photodynamic therapy, and laser ablation have also been proposed as potential treatment options for HPV+ intraepithelial squamous cell carcinoma in different locations such as penile, vulvar, and anal regions [45,81,82].

In a prospective randomized controlled trial (RCT), ablative and topical treatments for anal intraepithelial neoplasia (AIN) were compared in HIV-positive patients [83]. The therapeutic modalities included imiquimod for 16 weeks (three times per week), topical fluorouracil for 16 weeks (twice per week), or monthly electrocautery for 4 months, with the best response observed in the last group. Another prospective study reported an overall response rate of 66% of high-grade perianal and intra-anal squamous intraepithelial lesions in an HIV-infected group of patients treated with imiquimod in a five-day-per-week regime that could be extended up to 32 weeks [84]. For ablative treatments, laser therapy showed no significant difference in recurrence rates compared to electrocautery [85].

It is important to take into account that surgical smoke from electrocautery may be associated with an increased risk of occupational exposure to HPV. Although the severity of exposure differs according to certain factors, e.g., the ventilation or smoke evacuation system, the use of surgical masks, especially the N95 mask, should be enhanced to avoid air contact with virus particles [86,87].

### 6.2. Infiltrating HPV-Related SCC

Micrographic Mohs surgery (MMS) is a technique that was developed for the removal of skin tumors located in compromised areas in which saving tissue is essential and is associated with lower recurrence rates [88]. The technique usually involves the study of frozen tissue sections. However, for SCCs, a modified MMS technique (slow, 3D histology) with paraffin sections may allow routine immunohistochemical stainings and a better tumor visualization [89]. The histopathological study is carried out in tangential sections that determine the assessment of 100% of the tumor margins compared to the conventional vertical “bread-loaf” sections.

Genitalia and fingers are considered high-risk areas for the recurrence and a tissue-sparing technique such as MMS should be promoted in these anatomical areas [90,91]. In addition, although scarcely reported, MMS might allow tissue-sparing removal of oral dysplastic lesions and cSCC [92]. Although MMS seems to be the best option to treat nail cSCC, recurrence rates have been reported to be over 20% [48,53], which are clearly higher than the estimated rate of 3% with this technique in other locations. This may be explained by anatomical nail particularities, which may make the surgical approach more complex, as well as the immune privilege of the nail matrix where HR-HPV may persist and promote recurrences [48,49,50].

In SOTR, the modification of immunosuppression while reducing IS blood levels or introducing mTOR inhibitors should also be considered to reduce the risk of skin cancer [67]. In addition, it is essential that these patients receive lifestyle counseling and reduce exposure to additional risk factors for skin cancer such as sun exposure [65,67].

HPV+ oral SCC shows special sensitivity to treatment with chemotherapy and radiotherapy, being a broad consensus on the literature that indicates better survival rates free of disease for this group of patients [93]. This greater sensitivity to chemoradiation may be due to viral oncoproteins (E6, E7) impairing DNA repair, a robust immune response, fewer genetic mutations, and the typically younger patient profile, leading to improved treatment outcomes compared to HPV-negative tumors [93].

## 7. HPV Vaccination and SCC Development

Currently, there are three vaccines for HPV that have been approved by both the EMA and the FDA: the bivalent vaccine (Cervarix by GSK, Rixensart, Belgium), the quadrivalent vaccine (Merck, Sharp & Dohme (Merck & Co., Whitehouse Station, NJ, USA)), and the nonavalent vaccine (Merck, Sharp & Dohme (Merck & Co., Whitehouse Station, NJ, USA)) [94].

The World Health Organization (WHO) recommends HPV vaccination primarily for girls aged 9–14 years, ideally before they become sexually active. While all the vaccines are equally effective against HPV types 16 and 18, the nonavalent vaccine offers extra protection against HPV types 31, 33, 45, 52, and 58. The vaccines have shown high efficacy in young women who were HPV-seronegative before vaccination [94].

Safety data confirm that the HPV vaccines are safe, with the most common side effect being localized symptoms. Although the long-term effectiveness of the vaccines in reducing the incidence and mortality rates of HPV-related cancers remains to be fully seen, current data on cervical cancer support their effectiveness [95].

Recently, the use of vaccines has been proposed not just for prevention, but also for therapeutic purposes. The goal of a therapeutic vaccine against HPV is to stimulate in vivo virus-specific T cell responses targeting existing HPV infections and lesions. Since HPV oncoproteins E6 and E7 are only expressed in tumor cells, they serve as ideal targets for therapeutic vaccines. In most cases, therapeutic vaccines have targeted the E6 and E7 proteins, or a combination of both, as antigens. Therapeutic vaccines have been created using various platforms, including peptide- or protein-based vaccines, viral vector vaccines, bacterial vector vaccines, cell-based vaccines, and DNA- and RNA-based vaccines [94]. To date, there are no therapeutic vaccines that have irreversibly cured HPV-associated cancers. However, there are a few promising therapeutic vaccine candidates [94].

The evidence regarding the impact of vaccines on skin cancer is limited. Nichols and colleagues investigated the impact of the quadrivalent Gardasil vaccine on two patients with a history of multiple keratinocyte carcinomas [76,96]. Both patients experienced a reduction in the incidence of new SCC compared to their previous rates. Subsequently, the same researchers treated an immunocompetent woman in her 90s, who had numerous cSCCs on her leg, with the 9-valent HPV vaccine. She received two intramuscular doses of the vaccine six weeks apart, followed by intratumoral injections into three of the largest tumors. Over the next eight months, she received three additional intratumoral injections. Within two weeks after the second intratumoral dose, there was a noticeable improvement in the size and number of tumors. Eleven months after the initial intratumoral injection, the patient had no remaining cSCCs and sustained clinical remission for at least 24 months [96].

In summary, the effectiveness of vaccination in preventing HR-HPV infections is unquestionable. It has already been proven as lowering the incidence of certain cancers, such as cervical cancer. While it is likely that the vaccine also helps reduce skin cancers linked to HR-HPV, obtaining conclusive data on this may be challenging. Additionally, although it is premature to guarantee a cure through vaccination alone, therapeutic vaccines show promise as a future treatment option.

## 8. Conclusions and Future Directions

In conclusion, human papillomaviruses play a significant role in the carcinogenesis of a subset of mucosal and cutaneous squamous cell carcinomas. HPV is responsible for 39% of vulvar carcinomas, 47% of penile carcinomas, 90% of anal carcinomas, and 25% of the head and neck carcinomas, mainly related to α-HPV type 16. Several works have reported a better prognosis in mucosal carcinomas that are associated with HPV. Periungual SCCs are also frequently associated with HR-HPV.

β-HPV types, especially types 5 and 8, trigger the development of squamous cell carcinomas in epidermodysplasia verruciformis in synergy with ultraviolet radiation.

While UV-induced mutations promote cell cycle arrest and apoptosis of keratinocytes in immunocompetent patients, keratinocytes remain alive in immunosuppressed patients infected with β-HPV. This mechanism would be relevant at an early stage and dispensable for maintenance of cSCC (“hit-and-run” hypothesis).

An acquired form of EV, associated with HIV infection or iatrogenic immunosuppression, shows a similar clinical presentation. However, the role of HPV in immunocompetent patients is more controversial. However, α, β, and γ genera have been found in samples of actinic keratoses, invasive non-mucosal squamous cell carcinomas, and keratoacanthomas.

Regarding the treatment of in situ HPV-related SCC, topical immunomodulators such as imiquimod have not been demonstrated as achieving better responses than local ablative modalities such as electrocauterization. On the other hand, micrographic Mohs surgery should be recommended in patients with invasive mucosal squamous cell carcinomas, including those in the periungual area.

Finally, while the effectiveness of vaccination in preventing HR-HPV infections and subsequent genital squamous cell carcinomas is unquestionable, the therapeutic utility of these vaccines has yet to be demonstrated.

## Data Availability

Data are contained within the article. No new data were created in this article.

## Abbreviations

cSCC	Cutaneous squamous cell carcinoma
SCC	Squamous cell carcinoma
HPV	Human papillomavirus
EV	Epidermodysplasia verruciformis
URR	Upstream regulatory region
ORFs	Open reading frames
BD	Bowen’s disease
KA	Keratoacanthoma
AK	Actinic keratosis
OCSCC	Oral cavity squamous cell carcinoma
OPSCC	Oropharyngeal squamous cell carcinoma
ASCC	Anal squamous cell carcinoma
AIN	Anal intraepithelial neoplasia
MMS	Micrographical Mohs surgery
